# Inhalable exosomes to target cardiac repair

**DOI:** 10.20517/evcna.2024.81

**Published:** 2025-03-25

**Authors:** Ajit Magadum

**Affiliations:** ^1^Aging and Cardiovascular Discovery Center (ACDC), Lewis Katz School of Medicine, Temple University, Philadelphia, PA 19140, USA.; ^2^Center for Regenerative Medicine and USF Health Heart Institute, Department of Internal Medicine, University of South Florida, Tampa, FL 33602, USA.

**Keywords:** Inhalation, exosomes, cardiovascular disease, CD36, non-invasive delivery

## Abstract

A recent study introduces Stem Cell-derived Exosome Nebulization Therapy (SCENT), a novel, non-invasive strategy that delivers lung spheroid cell-derived exosomes via inhalation to promote cardiac repair after myocardial infarction. This approach improves cardiac function, reduces injury, and demonstrates translational potential in both small and large animal models, offering a promising avenue for cell-free, inhalable regenerative therapies.

Exosomes have a short half-life *in vivo*, necessitating repeated dosing to maintain therapeutic efficacy, especially in cardiovascular diseases (CVD), which remain a leading cause of death globally. A recent study published in *Circulation* introduces an innovative, non-invasive approach called Stem Cell-derived Exosome Nebulization Therapy (SCENT)^[1]^. This method uses exosomes derived from lung spheroid cells (LSCs) and delivers them via inhalation, offering a novel way to deliver exosomes to the heart for regenerative applications.

Myocardial infarction (MI), caused by restricted blood flow to the heart, leads to irreversible damage to cardiac tissue. Standard treatments do not repair the damaged tissue. Regenerative strategies, including stem cell therapies, have faced challenges such as poor stem cell retention, limited differentiation into cardiomyocytes, and issues with delivery methods. Exosomes, nanoscale extracellular vesicles (EVs) secreted by cells, have emerged as a promising alternative, carrying bioactive molecules like proteins, lipids, and RNA that mimic the paracrine effects of stem cells. It is well known that EVs derived from stem cells promote cardiac protection and improve cardiac function post-MI by inducing cell survival, cardiomyocyte proliferation, and angiogenesis, and inhibiting fibrosis, hypertrophy, and inflammation^[2-4]^.

Effectively delivering exosomes to the heart remains a significant challenge due to the limitations of conventional delivery methods. Intravascular and intraperitoneal administration result in rapid clearance, off-target accumulation, and low cardiac retention. Intramyocardial, pericardial, and epicardial injections, while more direct, are highly invasive, limit the feasibility of repeat dosing, and pose risks of cardiac damage. Similarly, intra-coronary delivery, although targeted, is transient and carries the risk of microvascular obstruction. The concept of inhaling biologics as a delivery route to the heart is novel, primarily due to the inherent difficulty of biologics crossing the air-blood barrier in the lungs. However, due to the lungs’ proximity to the heart, it presents an ideal route for distributing therapeutic agents. SCENT inhalation approach enables high bioavailability of exosomes at the site of cardiac injury, bypassing barriers like rapid clearance by the reticuloendothelial system or degradation in circulation. In Li *et al.*’s study, exosome therapy via LSCs was delivered through nebulization, directly targeting the lungs^[1]^. The study demonstrated that inhaled exosomes crossed the air-blood barrier and accumulated in ischemic cardiac tissue within 60 min, with reduced signals in the lungs suggesting continued exosome passage into circulation. Repeated dosing significantly increased exosome retention in the heart after 48 h. To ensure the bioactivity of the nebulized exosomes is preserved, appropriate potency assays and rigorous assessments of key nebulization parameters are essential.

This research demonstrated efficacy in both mouse and pig models of MI. In an acute MI model, mice treated with daily doses of LSC-derived exosomes for seven days showed significantly improved left ventricular ejection fraction (LVEF), reduced infarct size, and modulation of inflammation and tissue remodeling - key factors in MI progression compared to the PBS^[1]^. SCENT induced cardiomyocyte proliferation in the infarct zone and reduced cardiomyocyte apoptosis, resulting in a thicker left ventricular wall and enhanced contractile capacity. Similarly, in the pig model, which more closely mimics human physiology, substantial heart function improvements were observed, highlighting the translational potential of SCENT. All animals in the pig study survived until the 28-day endpoint with no significant abnormalities in hematology, liver, or kidney function, suggesting SCENT’s safety and its high potential for clinical application in MI^[1]^.

Exosomes have been shown to enhance cardiac function through various mechanisms, which are further explored in this study using the SCENT platform. RNA sequencing demonstrated that these exosomes are enriched with microRNAs (miRNAs) that promote heart repair. Notably, miR-100 was identified as the most prevalent, contributing to neovascularization and protecting cardiac tissues under stress by suppressing fatty acid uptake through the downregulation of CD36. The study’s bioinformatic analysis indicated that endothelial cells were the primary beneficiaries of this exosome therapy, with CD36 emerging as a critical gene influenced by the treatment. This research provides the first *in vivo* evidence that targeting CD36 in endothelial cells can mitigate cardiac injury post-MI, thereby highlighting new potential therapeutic targets in CD36 and associated pathways for enhanced cardiac repair^[1]^.

The findings of Li *et al.* could pave the way for the development of non-invasive, cell-free biologics, inhalable therapies for MI^[1]^. Emerging strategies in cardiac regenerative medicine rely on both systemic delivery or direct cardiac injections - such as RNA therapeutics (mRNA, miRNA, and non-coding RNAs), gene editing, Adeno associated virus (AAVs), and cell and tissue engineering. This approach offers several advantages: non-invasiveness, cell-free, repetitive dosing potential, and adaptability for exosomes from other cell types or engineered exosomes with specific therapeutic cargo. The next steps in clinical translation are the need for precise targeting of exosomes to cardiac cells, improving retention and bioactivity within the heart, understanding the complexity of exosome biology and content, and addressing regulatory hurdles^[5]^. Future studies are also necessary to explore the long-term effects of inhaled exosomes, and optimizing dosage and timing will be essential to maximize therapeutic benefits.

## DECLARATIONS

### Copyright

© The Author(s) 2025.
